# Public Attitudes Towards Moral Enhancement. Evidence that Means Matter Morally

**DOI:** 10.1007/s12152-017-9340-9

**Published:** 2017-07-27

**Authors:** Jona Specker, Maartje H. N. Schermer, Peter B. Reiner

**Affiliations:** 1 Department of Medical Ethics and Philosophy of Medicine, Erasmus Medical Center, University of Rotterdam, PO Box 2040, 3000 CA Rotterdam, The Netherlands; 2 National Core for Neuroethics, University of British Columbia, 2211 Wesbrook Mall, Vancouver, BC V6T2B5 Canada

**Keywords:** Moral enhancement, Moral bioenhancement, Experimental neuroethics, Public attitudes, Empathy, Mandatory interventions

## Abstract

To gain insight into the reasons that the public may have for endorsing or eschewing pharmacological moral enhancement for themselves or for others, we used empirical tools to explore public attitudes towards these issues. Participants (*N* = 293) from the United States were recruited via Amazon’s Mechanical Turk and were randomly assigned to read one of several contrastive vignettes in which a 13-year-old child is described as bullying another student in school and then is offered an empathy-enhancing program. The empathy-enhancing program is described as either involving *taking a pill* or *playing a video game* on a daily basis for four weeks. In addition, participants were asked to imagine either their own child *bullying* another student at school, or their own child *being bullied* by another student. This resulted in a 2 × 2 between-subjects design. In an escalating series of morally challenging questions, we asked participants to rate their *overall* support for the program; whether they would support *requiring* participation; whether they would support requiring participation of children who are *at higher risk* to become bullies in the future; whether they would support requiring participation of *all children* or even *the entire population*; and whether they would be willing to participate in the program themselves. We found that people were significantly more troubled by pharmacological as opposed to non-pharmacological moral enhancement interventions. The results indicate that members of the public for the greater part oppose pharmacological moral bioenhancement, yet are open to non-biomedical means to attain moral enhancement. [248 words].

**Electronic supplementary material:**

The online version of this article (doi:10.1007/s12152-017-9340-9) contains supplementary material, which is available to authorized users.

## Introduction

Moral competence is universally valued. Religious texts and the virtue ethics traditions all valorize the attainment of moral fluency. The Enlightenment brought its own contributions to the project, with deontology and consequentialism imparting ‘rational’ means of defining what it means to be a moral person [1].

Recently, a debate has emerged regarding the propriety of moral *bioenhancement* [2–5]. The suggestion is that we are on the cusp of understanding the neurological and genetic underpinnings of moral (and immoral) behaviour, and that we should use that knowledge to develop technologies that enhance human morality. However, what constitutes moral enhancement is highly contested [6, 7]: “clear and precise definitions of “moral enhancement” are not to be found; what has been called “moral” enhancement ranges from encouraging empathic concern to increasing personal responsibility all the way to heightening respect for global fairness” [8]. The debate is highly speculative. As the science of moral enhancement is “in its infancy”, neuroscientist Molly Crockett has warned to “be careful not to draw premature conclusions about potential avenues for moral bioenhancement” [9]. Most contentious of all has been the suggestion that moral bioenhancement ought to be compulsory.^1^ The debate [12–28] has been vigorous but is at somewhat of an impasse.

In earlier articles we therefore advocated a more focused debate on the potential domains for which moral bioenhancement interventions will most likely will be implemented [29, 30]. Similarly, Harris Wiseman has advocated a ‘practical-realities first’ approach to potential moral bioenhancement interventions, implying that speculation about moral bioenhancement should account for “the specific practical realities to be found on the ground level, which are not at all incidental but the very realities around which the abstractions of the debate must be made to shape themselves (not the other way around)” [4]. Given that the issue is of interest not just to philosophers but to the public at large, we explored public attitudes towards moral bioenhancement using both quantitative and mixed methods techniques.^2^

We carefully considered what sort of immoral behaviour would be best to evaluate in our studies. We previously found that the public was generally supportive of using pharmacological means to alter criminal behaviour so long as safety was assured [33], but empirical studies have found criminals to be outside of the circle of moral concern [34]. It is unclear whether members of the public are similarly supportive of biological interventions aimed at persons who engage in immoral yet legal behaviour. We therefore narrowed our focus to morally contentious behaviours that are not unlawful. We settled on bullying, an act that is generally condemned as immoral but usually does not cross the line to illegality.

Bullying is an act that is intended to harm, takes place repeatedly, and is characterized by a systematic abuse of the imbalance of power between the aggressor and target [35]. Bullying takes place in schools, between siblings, in prisons, and in the workplace [36], as well as online [37]. The observation that school bullying (both perpetration and victimization) predicts aggression and violence later in life has prompted calls for early prevention efforts [38, 39]. Although bullying per se is not the primary target of the moral bioenhancement debate, commentators have argued that “early childhood is probably the optimal starting point for moral enhancement” [40, 41].

Our objective was to test a range of issues that are central to the debate on moral bioenhancement. We hypothesize that the degree to which members of the public support an empathy-enhancing moral enhancement program depends on whether or not the means employed were pharmacological or non-pharmacological. We expect people to be less supportive of pharmacological than of non-pharmacological programs, even when safety and efficacy are held constant. In addition, we hypothesize that people are less supportive of pharmacological moral enhancement of their own children than they are of other people’s children. Second, we hypothesize that the degree to which respondents support these programs depends on whether they imagine themselves or someone outside their immediate circle of concern to participate. In other words, we expect that the distinction between self and other is relevant [42]. We expect that this distinction matters more for the pharmacological program than the pedagogical program. Finally, we hypothesize that the public is uncomfortable with mandating moral enhancement interventions, and particularly averse to mandatory *pharmacological* moral interventions.

## Methodology

### Experimental Methods

In order to explore attitudes of members of the public towards moral enhancement, we used the contrastive vignette technique [43]. The key outcome measure was the difference in group means between contrastive conditions rather than individual stated preferences. In addition to these quantitative measures, we also employed a novel mixed-methods design in which content analysis of free-response answers were quantitized and assessed in a contrastive fashion [44].

#### Vignette Design Strategy

Participants were presented with one (and only one) of several contrastive vignettes in which a 13-year-old child is described as bullying another student in school and then is offered an empathy-enhancing program. The vignettes were designed to be minimally contrastive, plausible, and to ensure that the results would be responsive to the hypothesis under consideration. Vignettes were analyzed using the Flesch-Kincaid Reading Ease and Grade Level readability tests, and we confirmed that a 15- to 21-year-old would easily understand the text of the vignettes.

One form of contrast involved the *means* of moral enhancement: the empathy-enhancing program was described as either involving *taking a pill* or *playing a video game* on a daily basis for four weeks [means: pharmacological or non-pharmacological]. Both programs were described as being equally safe and effective. We took pains to insure that the pharmacological moral bioenhancement was as innocuous as possible, describing it as a pill “based on the natural hormone oxytocin”, as we did not want to bias our results with off-putting interventions such as genetic modification or deep brain stimulation. A second form of contrast built into the vignettes compared the *closeness to the subject* of the individual who is under consideration for moral bioenhancement: participants were asked to imagine either their own child *bullying* another student at school, or their own child *being bullied* by another student at school [closeness to subject: other’s child or own child]. This resulted in a 2 × 2 between-subjects design.

In an escalating series of morally challenging questions, we asked participants to what degree they thought that it would be a good idea for the bully to participate in the program (question 1; anchors ranging from 0: bad idea to 100: good idea); to what degree they thought that it would be a good idea for the bully to be required to participate in the program (question 3); to what degree they thought that it would be a good idea for children, identified by a test to be at higher risk of being bullies in the future, to be required to participate in the program (question 5); to what degree they thought it would be a good idea for all children to be required in the program, given that the program increases empathy (question 6); to what degree they thought society would be better off if the general population was required to participate in the program (question 7; anchors ranging from 0: much worse off to 100: much better off); and to what degree they would be willing to participate in the program themselves (question 8; anchors ranging from 0: entirely unwilling to 100: entirely willing).

In the second part of the experiment, we asked participants to read a second vignette describing the same 13-year-old child bullying another student at school (either their child as bully, or their child being bullied), but this time is being required to participate in an the *alternative empathy-enhancing program*: participants who were initially presented with a program that involved taking a pill daily for four weeks, were now reading about an alternative program that involves playing a video game for four weeks – and vice versa. We presented them with the rating they gave in response to question 3, and asked them to what degree they thought that it would be a good idea for the bully to be required to participate in this alternative program.

We asked participants to explain the rationale for the answers they had given to questions 1, 3, and 9, in open response boxes (questions 2, 4, and 10), which were coded using Contrastive Quantitized Content Analysis [44]. A comprehension check probed whether participants remembered whether the vignette described their own child engaged in bullying of their own child being bullied at school. Finally, a pair of questions asked participants to *optionally* tell us whether they or their family members had been bullied or been bullies themselves.

The vignettes and questions can be found in the supplemental information. The University of British Columbia’s Behavioural Research Ethics Board approved the study.

#### Sample Population and Survey Format

Participants were recruited via Amazon’s Mechanical Turk [45, 46]. Participants provided informed consent, and after completion of the survey were compensated $0.50. Surveys were administered using Fluid Surveys, and survey responses were collected online on June 6, 2016.

#### Statistical Analysis

Data were analyzed using SPSS. Quantitative questions were analyzed using a two way independent Analysis of Variance (ANOVA) to identify significant main and interaction effects of the two independent variables on vignette measures. We analyzed significant effects with independent sample t-tests. Descriptive statistics were used to characterize the composition and properties of the sample. The datasets generated during and/or analysed during the current study are available from the corresponding author on reasonable request.

Answers entered into the free-response box in question 2 were analyzed using the mixed-methods strategy called Contrastive Quantitized Content Analysis (CQCA) [44]. The technique provides a mechanism for quantifying the content of participants’ answers and comparing them across contrastive conditions. Answers to the open-ended questions following questions 3 and 9 were included, but the data did not appear to be different so analysis of these responses is not presented here. In order to mitigate experimenter bias, we first randomized the full set of comments and blinded the coder to the particular experimental vignette read by the participant who offered a given comment. We then carried out traditional content analysis of the blinded comments, developing themes iteratively. An initial subset of ~50 comments was analyzed by two coders, and disagreements were discussed until consensus was reached. Each theme was treated as a binary variable, and each comment received either a 1 when the theme was present or 0 when the theme was absent. Once all comments were coded, the data were unblinded and the frequency with which any theme emerged in the comments was compared across contrastive conditions, with inferential statistics (Pearson Chi-Square) used to explore if any observed differences were meaningful. The code sheet used in contrastive quantitative content analysis for question 2 can be found in the supplemental information.

## Results

### Sample and Demographics

A total of 384 participants from the United States completed the survey; 91 participants failed the comprehension test resulting in a final sample of 293 respondents from 38 states and the District of Columbia (missing were: Alaska, Arizona, Maine, Mississippi, Nebraska, North Dakota, Oklahoma, South Dakota, Utah, West Virginia, Wyoming). The mean age was 35.7 years old (with a standard deviation of 11.8 years). Frequencies of sample demographics are summarized in Table 1 in Appendix B.

### Do Means Matter Morally?

An often-cited argument as to why we should explore the possibilities of moral bioenhancement is the lack of effectiveness of so-called traditional methods of moral enhancement, such as upbringing, socialization, and education [10, 47, but see 22, 48]. A related argument is that there are little principled differences between employing traditional and potential biomedical methods of moral betterment in terms of their ethical acceptability [49, 50]. David DeGrazia, for example, contends that many arguments against biomedical means also apply to traditional, non-biomedical means: “one should not inculcate moral values that are wrong, so how can a parent be sure that she or he is justified in providing a particular type of moral instruction? Also facing this challenge are public school teachers who attempt to inculcate in students certain moral virtues such as civility, respect for differences and concern for the poor” [47]. Likewise, according to the so-called ‘companions in innocence’ line of reasoning [51, 52], any principled argument against the attempt to making people morally better using genetic means, will also apply to educational and socialization efforts.

Other commentators have argued that there are in fact morally relevant differences between traditional and biomedical moral enhancement, for example because education is characterized by a fundamental moral equality between educator and educated, an equality that is lacking in the case of biomedical interventions aimed at reshaping the moral agency of others [17]. Or, along these same lines, because the distinction between (direct) biomedical (neurological, pharmacological) interventions and (indirect) traditional interventions tracks a more fundamental distinction between reason-responsive and reason-bypassing interventions, or between interventions that allow for active involvement of the person undergoing the intervention and those interventions that do not [53].

In this study, we hypothesized that members of the public would be less supportive of pharmacological than of non-pharmacological programs, even if in the programs were described as being equally safe and effective. Moreover, we expected that the public would be uncomfortable with mandating moral enhancement interventions and with mandatory *pharmacological* moral interventions in particular. We tested these hypotheses with an escalating series of morally challenging questions.

**Fig. 1:**
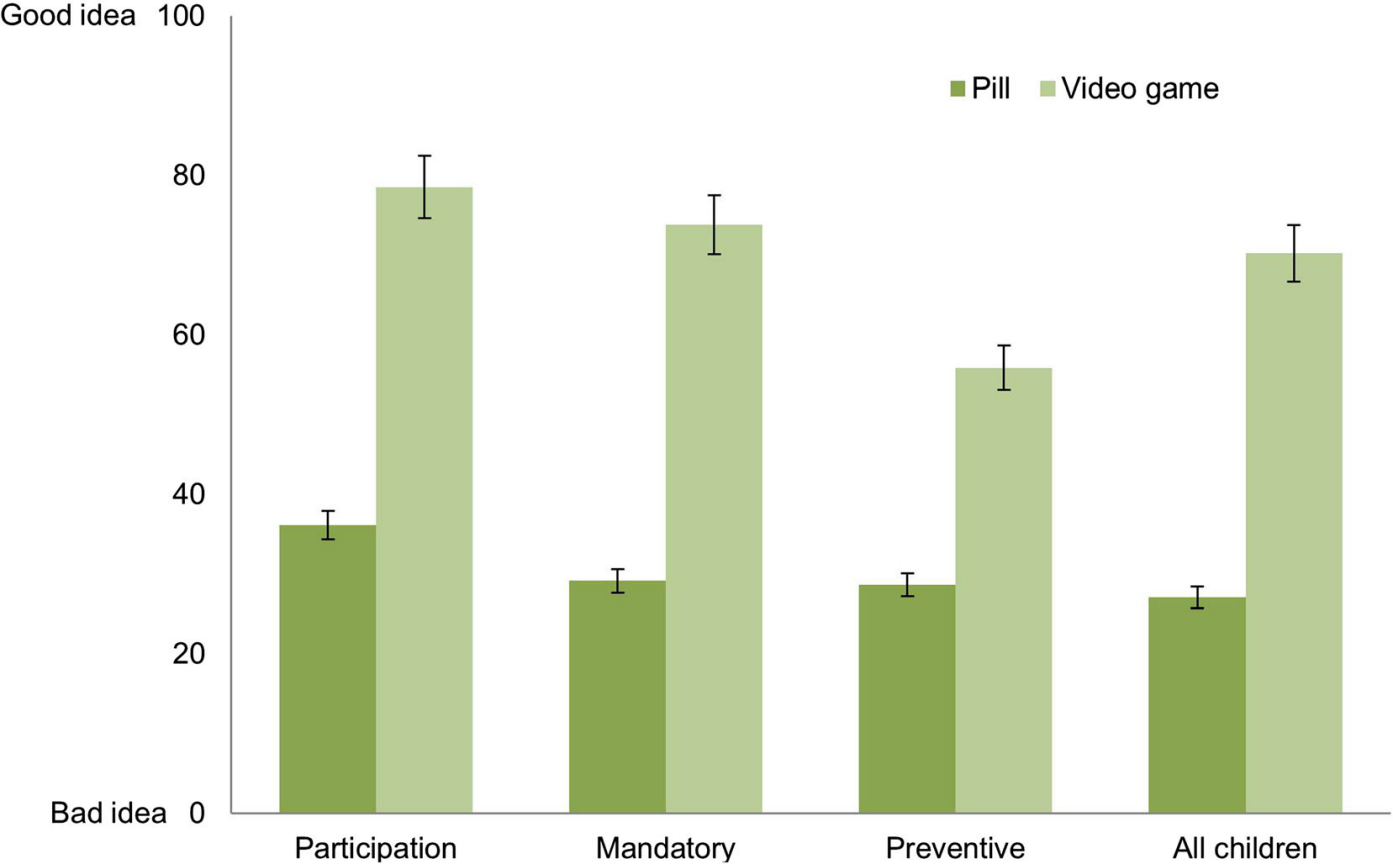
Mean ratings for participation, mandatory, and preventive empathy-enhancing anti-bullying programs and for empathy-enhancing programs for all children. Error bars represent 95% confidence intervals

The first question we posed asked whether it was a good idea for the bully to *participate* in the program as described in the vignette. A two way independent ANOVA showed a significant main effect of *version of program* (F(1289) = 141,57, *p* < 0.001). An independent sample t-test revealed that respondents were significantly more supportive of an anti-bullying program that involved playing a video game than one that involved taking a pill (M_diff_ = 42.39, 95%CI [35.32, 49.45], *p* < 0.001, *d* = 1.38) (Fig. 1, Participation).

We then asked respondents whether it would be a good idea for the bully to be *required* to participate in the program, using the same 101-point scale. A two way independent ANOVA showed a significant main effect of version of program (F(1289) = 147.26, *p* < 0.001). An independent sample t-test revealed that respondents were more supportive of a mandatory anti-bullying program that involved playing a video game than of a mandatory program that involved taking a pill; M_diff_ = 44.68, 95%CI [37.30, 52.05], *p* < 0.001, *d* = 1.39 (Fig. 1, Mandatory). Thus, respondents were significantly less supportive of a mandatory pharmacological than a mandatory non-pharmacological anti-bullying program.

Next, we asked respondents to rate their support for a mandatory *preventive* program for children who were identified as being at higher risk of being bullies in the future. A two way independent ANOVA showed a significant main effect of version of program on the support rates for the preventive anti-bullying program (F(1, 289) = 51.702, *p* < 0.001). An independent sample t-test revealed that respondents supported a preventive anti-bullying program that involved playing a video game more than a program that involved taking a pill; M_diff_ = 27.21, 95%CI [19.77, 34.65], *p* < 0.001, *d* = 0.84 (Fig. 1, Preventive). People were less supportive of empathy enhancement within the context of prevention of future immoral behaviour as compared to support for empathy enhancement in cases where immoral behaviour (bullying) has already manifested itself. This is of interest for debates on “public health approaches to preventing crime” and growing attention for early identification and prevention of antisocial behaviour [54–56].

Subsequently we asked respondents about empathy enhancing programs that go beyond bullying in schools, specifically whether it would be a good idea for *all children* (not just bullies or potential bullies) to be required to participate in the empathy-enhancing program. A two way independent ANOVA showed a significant main effect of *version of program* on the support rates for a mandatory empathy-enhancing program for all children (F(1289) = 131.005, *p* < 0.001). An independent sample t-test showed that respondents supported a mandatory preventive empathy-enhancing program for all children that involved playing a video game more than one that involved taking a pill; M_diff_ = 43.13, 95%CI [35.70, 50.57], *p* < 0.001, *d* = 1.33 (Fig. 1, All Children). Thus, respondents were significantly less supportive of requiring all children to participate in a mandatory pharmacological empathy-enhancing program compared to their support for required participation in a non-pharmacological program.

Taken together these results indicate that across a range of questions people were consistently more troubled by pharmacological than non-pharmacological moral enhancement interventions.

### The Distinction Between Self and Other

The second hypothesis driving this study is that people rate an empathy-enhancing program differently depending on whether they are imagining that their own child or someone else’s child is participating in the program. A previous study found that people employ double standards when thinking about the fairness of cognitive enhancement in situations where they would cognitively enhance themselves versus situations where others would do so: people perceive the same enhancing interventions as less ethically acceptable when other people use them than when they themselves use them [42]. Because a similar asymmetry may influence people’s reasoning about moral enhancement interventions, we explored the effects of vignettes which compared the distinction between self and other.

We tested this hypothesis in two ways. First, in contrastive versions of the vignettes respondents were asked to imagine either that their own child is bullying another student in school (own child), or that their own child is being bullied by another student in school (other’s child). Second, we compared responses to two questions in which we asked respondents about their support for a population-wide empathy-enhancing program (everyone else) and their willingness to participate in such a program themselves (self).

**Fig. 2:**
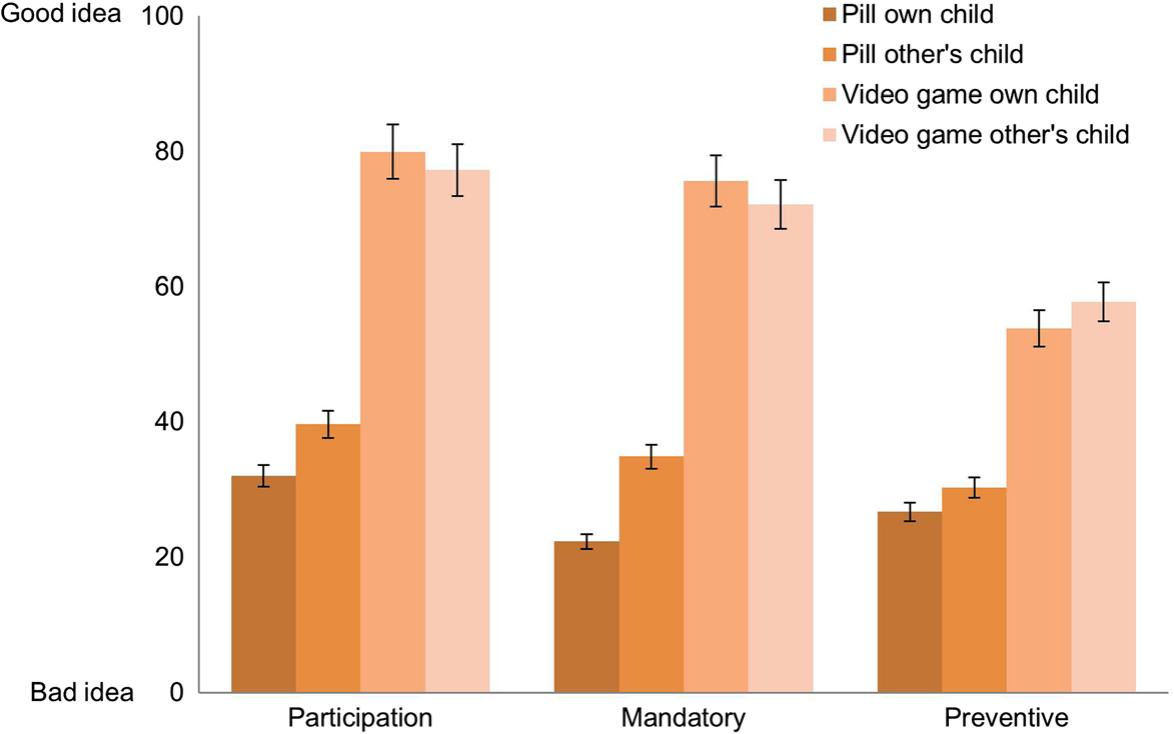
Mean rating for participation, mandatory, and preventive pharmacological and non-pharmacological empathy-enhancing anti-bullying programs for own child or other’s child. Error bars represent (95%) confidence intervals

We first analyzed the data to see if there was a difference between vignettes in which the empathy-enhancing program was to be administered to one’s own child who had been a bully (own child) versus those in which someone else’s child had been bullying the respondent’s child (other’s child). Two way independent ANOVAs demonstrate that there was no significant main effect of closeness to subject on the support rates for either *participation* (F(1289) = .451, *p* = .502), *mandatory* participation (F(1289) = 1.473, *p* = .226), or *preventive* approaches to the anti-bullying program (F(1289) = .994, *p* = .320) (Fig. 2).

There was a significant interaction (F(1289) = 4.4611, *p* = .033) between version of program and closeness to subject when the respondents were probed on children being *required* to participate in the program, indicating that the mean difference between other’s child and own child differs depending on whether the program involves a pill or a video game. Pairwise comparisons using an independent sample t-test revealed that people were more supportive of a mandatory empathy-enhancing program that involved taking a pill when they imagined the child participating in the program to be an other’s child rather their own child; M_diff_ = 12.55, 95%CI [1.77, 23.32], *p* = .023, *d* = 0.38 (Fig. 2, Mandatory).

In one of the first articles discussing moral enhancement, Thom Douglas argued that unlike other types of enhancement, moral enhancement primarily benefits others: “on any plausible moral theory, a person’s having morally better motives will tend to be to the advantage of others” [5]. Others have speculated about potential societal benefits of moral bioenhancement, arguing that, “they may, through contributing to civic virtue, help to secure the good functioning of our political institutions and processes. One way they could do this is by facilitating the dispositions towards cooperativeness and trust that plausibly underpin social solidarity” [57].

However, as “the advantages of moral enhancement may fall upon society rather than on those who are enhanced” [53], the need to balance potential risks to the one subjected to the program with benefits to others is arguably a central challenge when discussing *moral* enhancement. A fundamental question is how to weigh the interests and preferences of the individual and the interests of others (in view of public safety and managing public risk) [30].

**Fig. 3:**
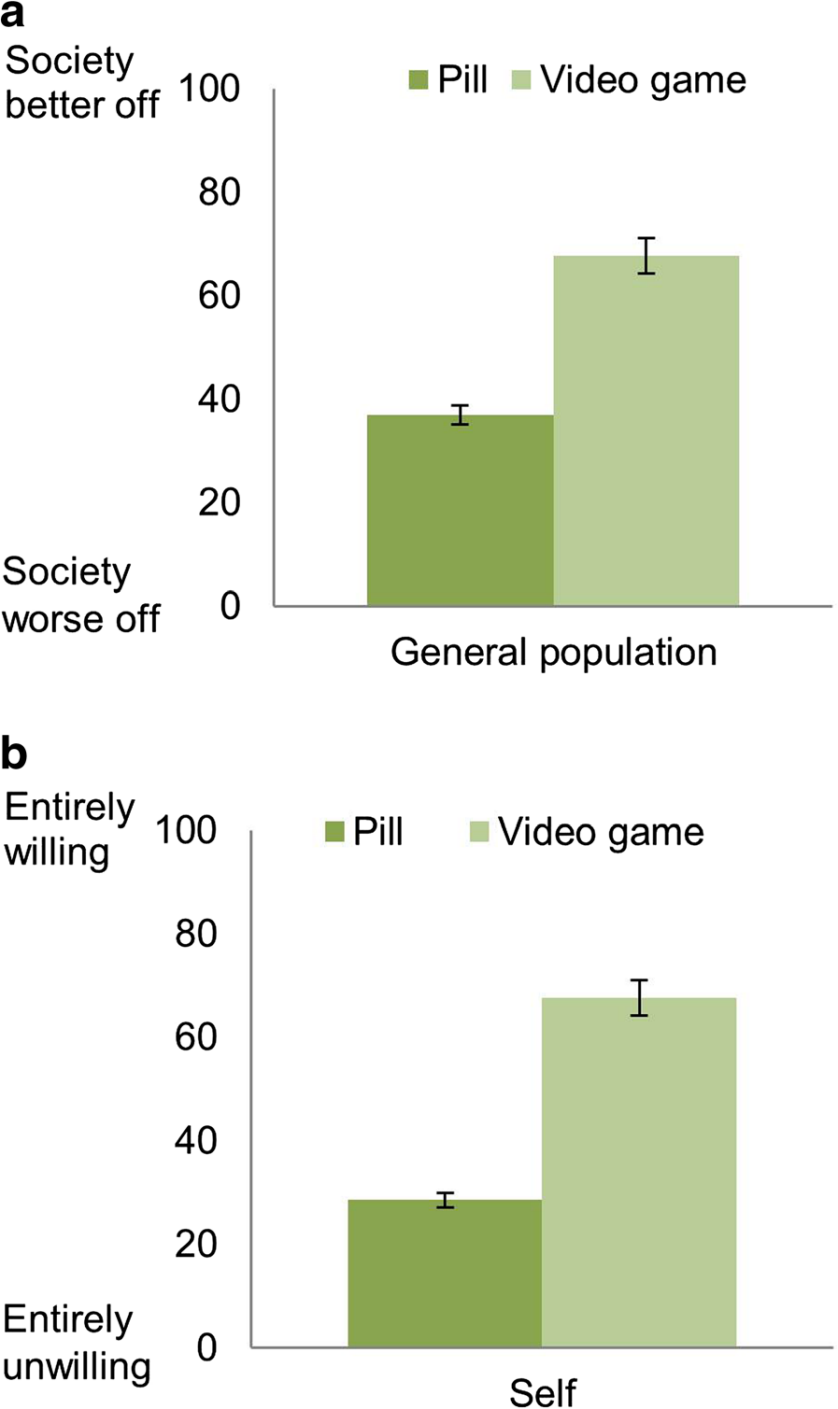
**a**, **b** Mean ratings for pharmacological and non-pharmacological empathy-enhancing programs, either with respect to the “degree society would be better off if the general population was required to participate in the program” and “degree you would be willing to participate in the program yourself.” Error bars represent (95%) confidence intervals

To address this, we asked participants whether they thought that society would be better off if the general population was required to participate in an empathy-enhancing program. As with the results presented earlier, a two way independent ANOVA showed a significant main effect of version of program on the support rates for a mandatory population-wide empathy-enhancing program (F(1289) = 67.808, *p* < 0.001). An independent sample t-test revealed that respondents supported a mandatory population-wide empathy-enhancing program that involved playing a video game more than one that involved taking a pill; M_diff_ = 30.72, 95%CI [23.27, 38.20], *p* < 0.001, *d* = 0.82 (Fig. 3a, General population). Subsequently, we asked whether respondents would be willing to participate in the empathy-enhancing program themselves. A two way independent ANOVA showed a significant main effect of version of program on willingness to participate (F(1289) = 93.432, *p* < 0.001). An independent sample t-test revealed that respondents were more willing to participate in an empathy-enhancing program that involved playing a video game than one that involved taking a pill; M_diff_ = 39.02, 95%CI [31.11, 43.51], *p* < 0.001, *d* = 1.13 (Fig. 3b, Self).

Finally, we asked whether participants had a history of bullying, either as perpetrators or victims of bullying. We found that 33.8% (*n* = 99) of the respondents indicated that they had been bullied in the past to such a degree that it interfered with their daily activities. 64.8% (*n* = 190) indicated that they hadn’t been bullied (to such a degree), and 1.4% (*n* = 4) chose to not answer this question. 7.8% (*n* = 23) of the respondents indicated that they had bullied in the past to such a degree that it interfered with someone else’s daily activities. 89.8% (*n* = 263) indicated that they hadn’t bullied someone else (to such a degree), and 2.4% (*n* = 7) chose to not answer this question. Age, gender, and whether participants had been bullied or were bullies themselves had no influence on any of our parameters.

### Reasons Offered for Attitudes Towards Empathy Enhancement

After rating the empathy-enhancing anti-bullying program described in the vignette on a sliding scale ranging from *good* to *bad idea* (question 1), participants were asked to explain why they answered as they did in a free-response format (question 2). Responses were analyzed using contrastive qualitative content analysis (see methods). The themes that emerged represented reasons that fell into four main categories: *Good idea, Bad idea, Ambivalent,* and *Appropriate reaction to bullying* (see appendix C for the code sheet).

#### Good Idea

**Fig. 4:**
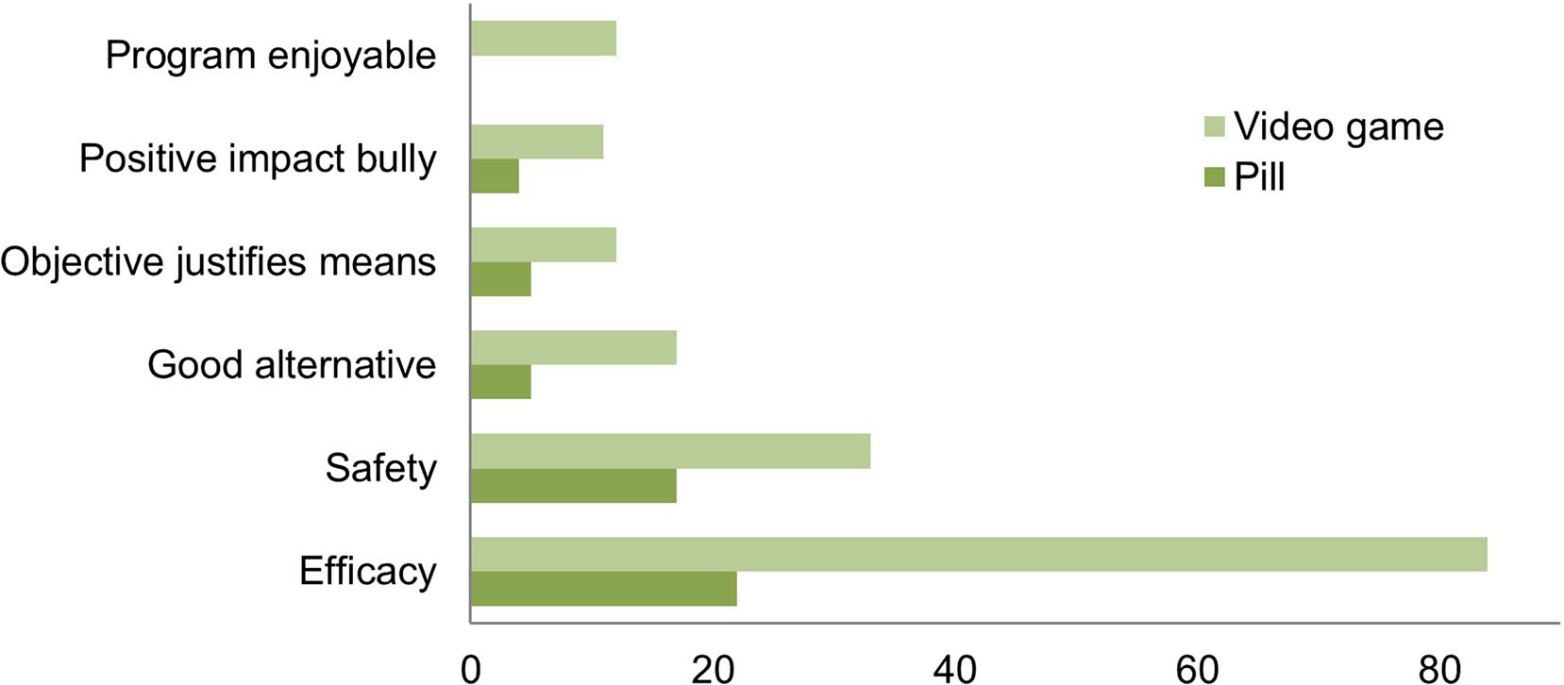
Reasons why the empathy-enhancing anti-bullying program is a *Good idea* (*n* = 230). Frequency of the theme as mentioned in comments (total number of comments, *n* = 620)

In the *Good idea* category, the following reasons in support of the empathy-enhancing anti-bullying program were given most frequently (cumulatively, across all versions of the vignette) (Fig. 4): the program’s EFFICACY (*n* = 106, 46.1%), the program’s SAFETY (*n* = 50, 21.7%), the notion that the program provides a GOOD ALTERNATIVE to other approaches (such as ignoring the problem or punishment) (*n* = 22, 9.6%), the idea that the OBJECTIVE JUSTIFIES MEANS (in spite of potential negative effects) (*n* = 17, 7.4%), the program’s POSITIVE IMPACT ON THE BULLY (as it will give him better chances in life, make him a better person, or will increase his flourishing) (*n* = 15, 6%), and the idea that the PROGRAM IS ENJOYABLE (and that this will motivate the bully to participate) (*n* = 12, 5.2%).

The program’s EFFICACY (*χ2* = 57.49, df = 1, *p* < 0.001) and SAFETY (*χ2* = 2.239, df = 1, *p* = 0.012), the notion that the program provides a GOOD ALTERNATIVE to current interventions (*χ2* = 7.166, df = 1, *p* = 0.007), and the notion that the PROGRAM IS ENJOYABLE (*χ2* = 12.598, df = 1, *p* < 0.001) were significantly more commonly mentioned in support of the non-pharmacological than the pharmacological program. No difference was found between version of the program and the following reasons in support of the program: the notion that the OBJECTIVE JUSTIFIES MEANS (*χ2* = 3.111, df = 1, *p* = 0.078), the program’s POSITIVE IMPACT on the BULLY (*χ2* = 3.494, df = 1, *p* = 0.062). We found no relationship between reasons in support of the program and closeness to subject (own child or other’s child), even when accounting for version of the program (pill or video game).

#### Bad Idea

**Fig. 5:**
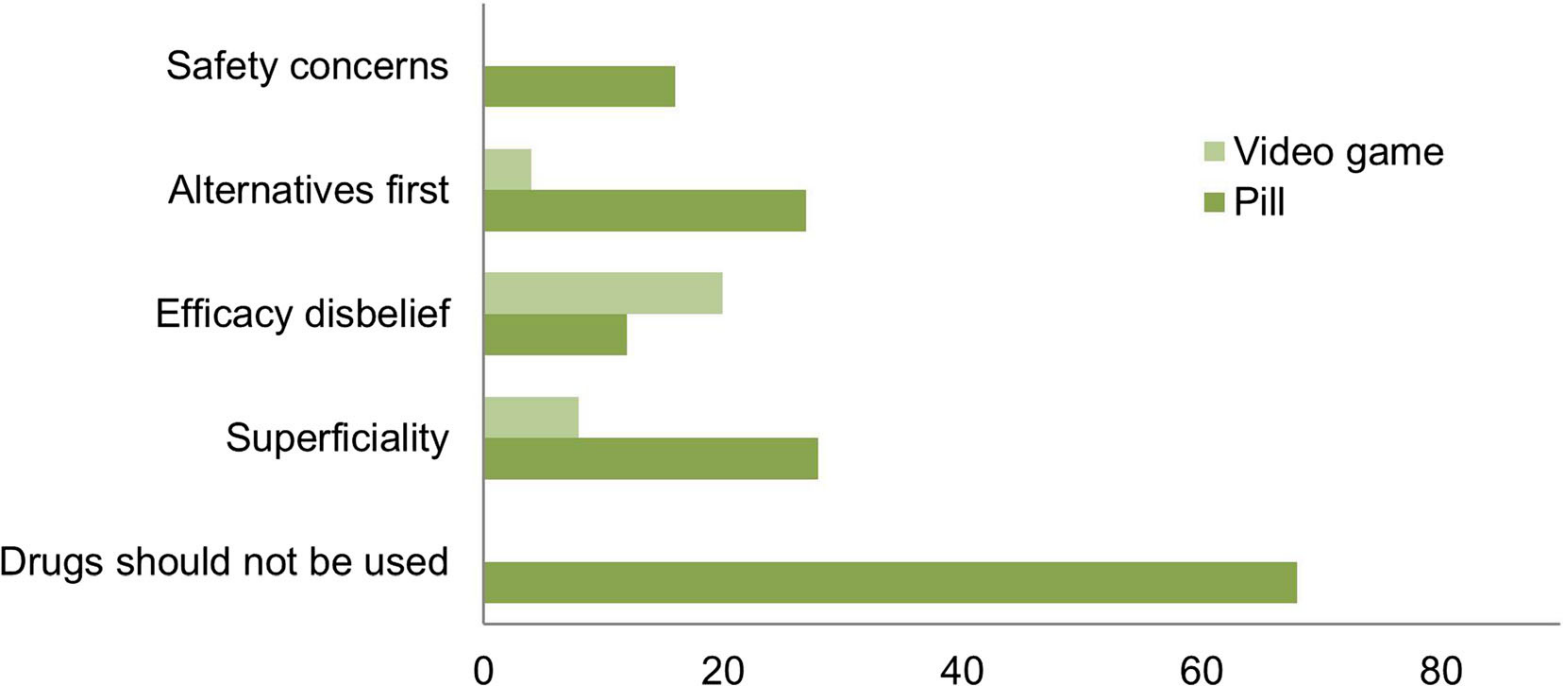
Reasons why the empathy-enhancing anti-bullying program is a *Bad idea* (*n* = 202). Frequency of the theme as mentioned in comments (total number of comments, *n* = 620)

In the *Bad idea* category, the following reasons against the program were given most frequently (Fig. 5): the notion that DRUGS SHOULD NOT BE USED (because they are artificial, because behavioural problems should not be remedied by taking drugs, or because there is nothing medically wrong with the child) (*n* = 68, 33.7%), the program’s SUPERFICIALITY (as it addresses symptoms and not underlying causes, or because it offers no durable solution) (*n* = 36, 17.8%), EFFICACY DISBELIEF (disbelief that the program will effectively lower the bullying, or disbelief that increasing empathy will lower the bullying) (*n* = 32, 15.8%), the notion that ALTERNATIVE APPROACHES should be tried FIRST (and that the program should be a last resort) (*n* = 31, 15.3%), SAFETY CONCERNS (concerns about side-effects, long-term effects, and concerns about addiction) (*n* = 16, 7.9%).

The following concerns were more commonly brought up against the *pharmacological program* compared to the non-pharmacological program: the notion that DRUGS SHOULD NOT BE USED (χ2 = 87.949, df = 1, *p* < 0.001), the SUPERFICIALITY of the program (*χ2* = 12.513, df = 1, *p* < 0.001), the notion that ALTERNATIVES should be tried FIRST (*χ2* = 18.909, df = 1, *p* < 0.001), and SAFETY CONCERNS (χ2 = 16.809, df = 1, *p* < 0.001). No difference was found between versions of the program for EFFICACY DISBELIEF (*χ2* = 2.307, df = 1, *p* = 0.129).

Chi-squared tests were performed to determine whether there was a relation between reasons why respondents rated the program as a bad idea and their imagined closeness to the bully (own child or other’s child). We found no significant relationship between closeness to subject (own child or other’s child) and particular reasons provided against the program without stratifying for version of program (pharmacological or non-pharmacological). However, the notion that ALTERNATIVES should be tried FIRST (*χ2* = 5.930, df = 1, *p* = 0.015) and SAFETY CONCERNS (*χ2* = 36.886, df = 1, *p* = 0.049) were more commonly mentioned as reasons against the program when the program described in the vignette was a *pharmacological* program intended for one’s *own child*. Moreover, we found that comments by respondents imaging their *own child* participating in a *pharmacological program* were more often coded as AMBIVALENT (*χ2* = 4.848, df = 1, *p* = 0.028).

Respondents appear to perceive a difference in safety between non-pharmacological and pharmacological programs, in spite of the fact that the empathy-enhancing program was presented as equally safe and effective in contrasting versions of the vignettes. When confronted with a non-pharmacological program, many respondents indicated that the program’s safety was an important reason for their support of the program, whereas respondents who had read a vignette that described a pharmacological program mentioned concerns about safety as a reason against the program. Moreover, concerns about safety were more commonly mentioned in response to pharmacological programs intended for own child than for other’s child.

#### Appropriate Reactions to Bullying

**Fig. 6:**
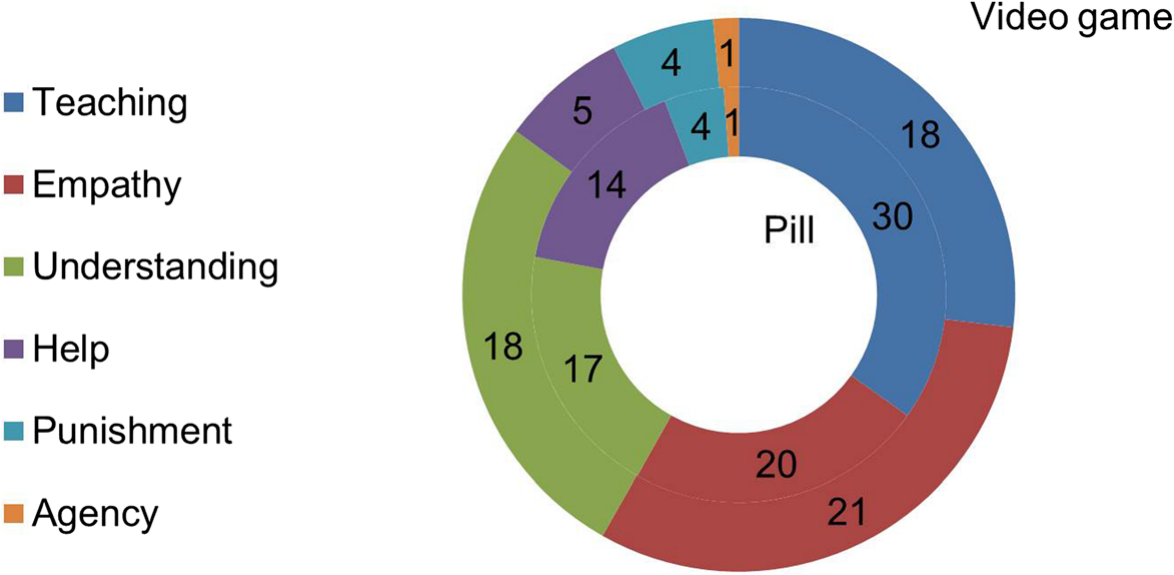
Appropriate reactions to bullying (*n* = 153). Frequency of the theme as mentioned in comments (total number of comments, *n* = 620). (Inner circle = pill, outer circle = video game)

In their answers, respondents oftentimes not only provided reasons for or against the program, but also explained what they considered to be an appropriate reaction to bullying. Appropriate strategies to alleviate bullying that were mentioned by respondents were: TEACHING (*n* = 48, 31.4%), EMPATHY (*n* = 41, 26.8%), UNDERSTANDING (*n* = 35, 22.9%), HELP (*n* = 19, 12.4%), PUNISHMENT (*n* = 8, 5.2%), and moral AGENCY (*n* = 2, 1.3%) (Fig. 6).

**Fig. 7:**
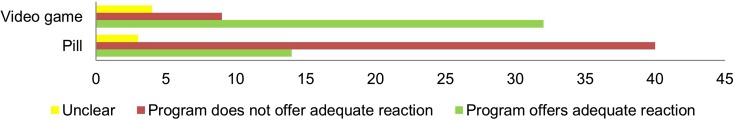
Does the program provides an adequate reaction to bullying or not (*n* = 153). Frequency of the theme as mentioned in comments (total number of comments, *n* = 620)

Moreover, respondents frequently made explicit whether they thought the program as described in the vignette indeed consisted of the strategy to alleviate bullying that they preferred or found to be most promising (Fig. 7). Chi-square tests were performed to determine whether there was a relationship between what respondents considered an appropriate reaction to bullying, and version of the program (pill or video game). Respondents more commonly reasoned that the bully needed HELP when confronted with a pharmacological rather than a non-pharmacological program (*χ2* = 4.493, df = 1, *p* < 0.034) (data not shown). We found no significant relation between version of program and respondents indicating that the bully needed TEACHING, EMPATHY, UNDERSTANDING, PUNISHMENT, or AGENCY.

When the program described in the vignette consisted of playing a video game, respondents more commonly expressed that they considered the program an adequate reaction to bullying (*χ2* = 8.502, df = 1, *p* = 0.004), while when the program involved taking a pill, respondents more commonly indicated that they did not consider the program an adequate reaction to bullying (*χ2* = 23.298, df = 1, *p* < 0.001) (Fig. 7).

In conclusion, the difference between self and other (either between one’s own child and an other’s child, or between the general population and oneself) influences the public’s support for moral enhancement. For the pharmacological empathy-enhancing program, respondents were more critical when they imagined their own child rather than an other’s child to be the one subjected to the program. They more often expressed ambivalence, mentioned safety concerns, and argued that alternatives should be tried first. And as regards the pharmacological program, respondents were more open to requiring the general population to participate in the program, but were less willing to participate themselves. However, the difference between self and other was largely absent for vignettes describing a non-pharmacological program, suggesting that this distinction is morally salient only when safety and risk concerns come into play.

## Discussion

This study provides empirical evidence that *means matter morally*. For when it comes to moral enhancement, members of the public generally eschew pharmacological moral bioenhancement yet are open to non-biomedical means to attain moral enhancement. Both the quantitative and the qualitative data confirm that the public disapprove of biomedical interventions for moral enhancement. These findings were confirmed convincingly across a range of questions and in all versions of the vignettes.

These findings are in line with previous research demonstrating a considerable bias against or mistrust of “pills” in general [58–60]. The added value of this study is that it sheds light on what kind of reasons members of the public have for their dislike of pharmacological interventions. Interestingly, when reflecting on the non-pharmacological program, many respondents explicated their support by reference to the fact that the program is described as safe and effective in the vignette. However, respondents who had read a vignette about the pharmacological program were often sceptical about the program’s safety and effectiveness, even though the program was described as equally safe and effective in the vignette. Again, as might be expected based on earlier research, respondents argued that pills are bad because they are artificial or unnatural, expressed concerns about safety and undue medicalization (over-medicalization) of behavioural problems [61, 62]. In addition however, many respondents reasoned that the pharmacological program offered no “real” solution to the problem; they were sceptical about the long-term effectiveness of the program and expressed concerns about the pharmacological program being too superficial and not adequately addressing underlying causes of the bullying behaviour. With the video game, respondents were more optimistic about its lasting effects.

Moreover, many respondents explained that the reason why they did or did not support the empathy-enhancing program in the vignette was related to whether or not they were under the impression that the program offered an appropriate pedagogical response to the problem behaviour (bullying). This might be interpreted as an indication that the public values fostering in children deeper understanding of and insight into why certain behaviour is morally wrong rather than mere conformity to (moral) rules. The public appears concerned not only about effectiveness (will an intervention reliably lower the immoral behaviour?) but also about whether the one participating in the program will, as a result of the intervention, have *learned* something on a deeper level and in the longer run.

One important consideration in interpreting these results is the fact that the scenario in our vignettes concerns bullying by a 13-year-old child. People might be particularly resistant to giving pharmacological substances to children, and different ethical considerations may come into play, for example about the responsibility of parents and schools. However, comments given in the open-response boxes indicate that the concerns respondents have go beyond mere ‘pills are bad’-considerations. Moreover, next to empathy enhancement in children, we also asked respondents about their support for empathy enhancement for the general population and for themselves.

Concerns about the importance of *doing the right thing, period* as opposed to *doing the right thing for the right reasons* are also raised in the ethical debate on moral bioenhancement. Douglas [25] discusses so-called “superficiality concerns” about forms of non-cognitive or reason-bypassing interventions that directly alter emotions. These interventions can be considered *brute* as opposed to *deliberate* [63], because they directly alter emotions without requiring the exercise of deliberative faculties, such as “moral reasoning, introspective reflection on one’s moral failures, or calm moral discussion with others” [64]. Douglas argues that these kinds of interventions are sometimes permissible [5]; Persson and Savulescu even argue that moral bioenhancement might be morally obligatory [11]. Other commentators disagree and reason that pharmacological or neuro-scientific interventions fail to produce *deep* moral understanding and *deep* moral improvement because these kinds of interventions fail to provide any moral *content.* 3

Harris maintains that to be a moral agent is to consider moral reasons for action. He argues that direct, reason-bypassing interventions “might well take the conduct of the affected individual beyond moral review and certainly out of the realm of things that might be right all things considered” [66]. Jotterand stresses that on a virtue ethical account, both moral emotions and moral reasoning are essential for autonomy and true moral agency:

“On my analysis, I conclude that moral neuroenhancement is unlikely to morally enhance people in the true meaning of the word. The development of neurotechnologies will allow us to control moral emotions but not to generate any content for moral reasons for actions. Without a systematic reflection on the nature of the good, the right and the just, one would end up, using MacIntyre’s language, in bad character because of intellectual blindness. Moral agency requires understanding and the formation of right moral emotions. (…) The hope of controlling human moral emotions is insufficient for the formation of virtuous people. Moral agents are not engineered but trained through the development of a vision of the good life and an understanding of human flourishing.” [67]

These comments suggest that at least some philosophical positions in the ongoing debate align with public opinion. Indeed, the reasons participants offered in their free responses reflect to large extent key themes discussed in the neuroethics literature.^4^ Furthermore, it is one thing to argue eloquently for or against the propriety of such things as mandatory biomedical moral enhancement and quite another to accept that mandate for yourself, or even more importantly for your children. Except under the auspices of a totalitarian state, the prospect of widely disseminating moral bioenhancement depends entirely upon the accession of the public. Our data demonstrate quite clearly that support for such a project is absent, even though advancing the moral skills of the populace enjoys widespread support.

## Electronic Supplementary Material

ESM 1 (PDF 603 kb)

## Footnotes

1 In their first publication on moral enhancement, Ingmar Persson and Julian Savulescu famously argued: “If safe moral enhancements are ever developed, there are strong reasons to believe that their use should be obligatory, like education or fluoride in the water, since those who should take them are least likely to be inclined to use them” [10], and in subsequent publications they have reaffirmed this position: “we do not rule out that moral bioenhancement could be justifiably imposed without the informed consent of the subjects” [11].

2 Previous studies on attitudes towards enhancement of a range of different traits suggested that people are least open to enhancing traits they believe to be more fundamental to the self/ identity, which included morally relevant traits (such as empathy and kindness) [31, 32].

3 Baertschi [65] explains that these disagreements can partly be attributed to different (i.e. cognitivist versus sentimentalist) meta-ethical positions.

4 We thank an anonymous reviewer for pointing this out.
