# Supplementary material for: Public Attitudes Towards Moral Enhancement. Evidence that Means Matter Morally
Source: Neuroethics. 2017 Jul 27;10(3):405–17. doi: 10.1007/s12152-017-9340-9 (PMC5569135; doi:10.1007/s12152-017-9340-9)
Supplement: Supplementary file 1 — (PDF 603 kb) [file 12152_2017_9340_MOESM1_ESM.pdf]

Supplementary material

Appendix A: Vignettes and questions

(Data gathered on Monday June 6, 2016)

**PHARMACOLOGICAL/ OTHER'S CHILD BULLIES**	**PHARMACOLOGICAL/ OWN CHILD BULLIES**
Imagine that your 13-year-old child was being bullied by another student at school. The school has a program that has been shown to be effective in reducing bullying in carefully carried out studies.<br>The program involves the following: over the course of 4 weeks, each day the bully takes a pill that increases empathy for others. The pill is based on the natural hormone oxytocin, and improves the bully's ability to understand what other people are feeling. Studies have shown that the program reduces bullying by 40%, with no side effects. The reduction in bullying persists for 6 months after the program is complete. [104 words]	Imagine that your 13-year-old child was bullying another student at school. The school has a program that has been shown to be effective in reducing bullying in carefully carried out studies.<br>The program involves the following: over the course of 4 weeks, each day the bully takes a pill that increases empathy for others. The pill is based on the natural hormone oxytocin, and improves the bully's ability to understand what other people are feeling. Studies have shown that the program reduces bullying by 40%, with no side effects. The reduction in bullying persists for 6 months after the program is complete. [102 words]
**NON-PHARMACOLOGICAL/ OTHER'S CHILD BULLIES**	NON-PHARMACOLOGICAL/ OWN CHILD BULLIES
Imagine that your 13-year-old child was being bullied by another student at school. The school has a program that has been shown to be effective in reducing bullying in carefully carried out studies.<br>The program involves the following: over the course of 4 weeks, each day the bully plays a video game that increases empathy for others. The video game is based on best educational practices, and improves the bully's ability to understand what other people are feeling. Studies have shown that the program reduces bullying by 40%, with no side effects. The reduction in bullying persists for 6 months after the program is complete. [105 words]	Imagine that your 13-year-old child was bullying another student at school. The school has a program that has been shown to be effective in reducing bullying in carefully carried out studies.<br>The program involves the following: over the course of 4 weeks, each day the bully plays a video game that increases empathy for others. The video game is based on best educational practices, and improves the bully's ability to understand what other people are feeling. Studies have shown that the program reduces bullying by 40%, with no side effects. The reduction in bullying persists for 6 months after the program is complete. [103 words]

Q1. To what degree do you think that it would be a good idea for the bully to participate in a program like the one described above?

0... 100

Bad idea Good idea

Q2. Please tell us why you answered as you did.

Q3. To what degree do you think that it would be a good idea for the bully described above to be required to participate in the program?

0... 100

Bad idea Good idea

Q4. Please tell us why you answered as you did.

Q5. If there was a reliable test that identified children who are at higher risk of being bullies in the future, to what degree do you think that it would be a good idea if they would be required to participate in the program?

0... 100

Bad idea Good idea

Q6. Given that the program increases empathy, to what degree do you think it would be a good idea for all children (not just bullies or potential bullies) to be required to participate in the program?

0... 100

Bad idea Good idea

Q7. If this program increased empathy in everyone, to what degree would you think that society would be better off if the general population was required to participate in the program?

0... 100

Much worse off Much better off

Q8. To what degree would you be willing to participate in the program yourself?

0.. 100

Entirely unwilling | Entirely willing

Q9.

**There is an alternative program that is equally effective in increasing empathy for others, but involves the following: over the course of 4 weeks, each day the bully takes a pill that increases empathy for others. The pill is based on the natural hormone oxytocin, and improves the bully's ability to understand what other people are feeling. Once again, studies have shown that the program reduces bullying by 40%, with no side effects. The reduction in bullying persists for 6 months after the program is complete.**	**There is an alternative program that is equally effective in increasing empathy for others, but involves the following: over the course of 4 weeks, each day the bully plays a video game that increases empathy for others. The video game is based on best educational practices, and improves the bully's ability to understand what other people are feeling. Once again, studies have shown that the program reduces bullying by 40%, with no side effects. The reduction in bullying persists for 6 months after the program is complete.**

Given that this alternative program exists, to what degree do you think that it would be a good idea for the bully described above to be required to participate in this alternative program?

[Your rating for the original program was XX.]

0.. 100

Bad idea | Good idea

Q10. Please tell us why you answered as you did.

Q11. In the vignette described above you were asked to imagine that

Your 13-year-old child was being heavily bullied by another student at school

Your 13-year-old child was heavily bullying another student at school

Q12. Have you or any of your family members ever been so substantially bullied that it interfered with your daily activities? [optional question] Yes / No

Q13. Have you or any of your family members ever been so much of a bully that it interfered with someone else's daily activities? [optional question] Yes / No

Appendix B: Sample demographics

Table 1. Sample demographics, *n* = 293

Characteristic	*% (n)*
Gender	
Male	50.9% (149)
Female	49.1% (144)
Age (groups)	
18-30	41.6% (122)
31-45	38.9% (114)
46-65	18.1% (53)
>65	1.4% (4)
Education (groups)	
Some high school	0.3% (1)
High school diploma	13.0% (38)
Some college	31.1% (91)
College degree	43.0% (126)
Some post-graduate	2.7% (8)
Post-graduate degree	9.9% (29)

Appendix C: Code sheet

**Prefer Use**	**Safe:** this code captures comments that the program is safe<br><br>**Effective:** this code captures comments that the program is effective or that mention the degree of effectiveness of the program<br><br>**Objective justifies means:** this code captures comments that in spite of potential negative effects, the program is justified or worth trying given the negative effects of bullying<br><br>**Positive Impact bully:** this code captures comments that the program will likely benefit the child that is bullying (will give him more chances in life, will make him a better person, will increase his flourishing).<br><br>**Positive Impact victims:** this code captures comments that the program will likely benefit the child that is bullied.<br><br>**Program is enjoyable:** this code captures comments that the bully likely would enjoy the program, be motivated to participate in it, and that this would make it more likely that (s)he will stick with it<br><br>**Good alternative to present approaches:** this code captures comments that the program provides a good or viable alternative to present approaches to bullying (such as ignoring the problem, or punishment), and that the bullying behaviour needs to be addressed, that something needs to be done to stop the bullying<br><br>**Pill not bad:** this code captures comments that there is nothing (inherently) wrong with taking a pill
**Against Program because**	**Safety Concerns:** this code captures comments about side effects and safety concerns, about risk of becoming dependent or addicted, and comments that express disbelief that the program has no side effects, or no side effects in the long term.<br><br>**Efficacy Disbelief:** this code captures comments that explicitly express disbelief that the program will be effective in reducing bullying (for example because 'once a bully, always a bully', or because increase in empathy will not decrease bullying).<br><br>**Drugs should not be used to remedy behavioural problems in children/ concerns about medicalization of normal child behaviour:** this code captures comments that behavioural

	problems should not be remedied by taking drugs, comments that there is nothing medically wrong with the child/ no medical condition/ no acute medical condition, general comments that drugs are bad, wrong, or should not be used, as well as concerns that the program is artificial or not natural (e.g. chemical feelings, hormones)<br><br>**Need for (more) permanent and less superficial solution:** this code captures concerns that the program offers no permanent or durable solution to the bullying, and/ or that the program should not sidestep the real issues, that it should address underlying causes, not symptoms, as well as comments that express disbelief that the program will be effective after six months, after the program stops, in the long term, etc.<br><br>**Alternatives First/ last resort:** this code captures comments that other ways of dealing with the bullying child need to be tried first, as well as comments that the program should be a last resort, or only be used for the most grave instances.<br><br>**Autonomy:** this code captures comments that the bully should have the right to consent to the program, or that the program infringes on his autonomy<br><br>**Identity, personality, fundamental changes to the self, diversity:** this code captures comments that a person should not be asked, or forced to change his personality, as well as comments that ask who should decide what kind of changes in personality would in fact be desirable, and comments that people differ and that diversity in personality traits should be embraced instead of eliminated.<br><br>**Parents should decide:** this code captures comments that parents should be consulted, and consent to the program<br><br>**Doctor should be consulted:** this code captures comments that a doctor needs to be consulted
**Ambivalent**	**Ambivalent:** When it was overtly stated one or more reasons for and against the program, or that the commenter was unsure.
**Appropriate reaction to bullying/ The problem underlying bullying that needs to be addressed is:**	**Bully needs to be taught:** this code captures comments that the bullying child needs to be taught<br><br>**Bully needs to understand:** this code captures comments that the bullying child needs to understand why bullying is wrong<br><br>**Bully needs to empathize with victims:** this code captures

	comments that the bully needs to empathize with victims, and feel and understand how his actions hurt other people<br><br>**Bully needs punishment:** this code captures comments that the bullying child needs punishment<br><br>**Bully needs help:** this code captures comments that the bully needs help, that he likely has problems, is a victim himself<br><br>**Bully needs to take responsibility/ agency:** this code captures comments that the bully needs to bear the consequences of his (past) behaviour, needs to take responsibility for (future) behaviour
**Program provides an appropriate reaction to bullying**	**Program provides:** this code captures comments that program is an adequate reaction to bullying<br><br>**Program does not provide:** this code captures comments that the program is not an adequate reaction to bullying<br><br>**Unclear whether program provides:** this code captures comments where it is unclear whether the program provides an adequate reaction to bullying

Appendix D: Overall coding results

	All	Pill	Video Game	Pill own child	Pill other's child	Video game own child	Video game other's child	Own child	Other's child
GOOD Efficacy	106	**22**	***84**	9	13	39	45	48	58
GOOD Safety	50	**17**	***33**	9	8	15	18	24	26
GOOD Good alternative	22	**5**	***17**	1	4	6	11	7	15
GOOD Objective justifies means	17	5	12	2	3	4	8	6	11
GOOD Positive impact bully	15	4	11	0	4	8	3	8	7
GOOD Other									
GOOD Program enjoyable	12	**0**	***12**	0	0	7	5	7	5
GOOD Positive impact victim	5	3	2	1	2	1	1	2	3
GOOD Pill not bad	3	3	0	1	2	0	0	1	2
BAD Drugs are bad	68	***68**	**0**	36	32	0	0	36	32
BAD Superficiality	36	***28**	**8**	17	11	1	7	18	18
BAD Alternatives first	31	***27**	**4**	***18**	**9**	1	3	19	12
BAD Efficacy disbelief	32	12	20	3	9	12	8	15	17
BAD Safety concerns	16	***16**	**0**	***11**	**5**	0	0	11	5
BAD Other									
BAD Fundamental changes to self	5	1	4	1	0	2	2	3	2
BAD Parents should consent	7	5	2	3	2	0	2	3	4
BAD Autonomy	3	2	1	1	1	0	1	1	2
BAD Doctor should be consulted	4	4	0	3	1	0	0	3	1
Ambivalent	35	17	18	***12**	**5**	9	9	21	14
Overall FOR	139	**31**	***108**	**7**	***24**	52	56	59	80
Overall AGAINST	111	***94**	**17**	45	49	7	10	52	59

Overall UNCLEAR	9	6	3	4	2	2	1	6	3
NEED FOR Teaching	48	30	18	15	15	8	10	23	25
NEED FOR Understanding	35	17	18	5	12	11	7	16	19
NEED FOR Empathy	41	***20**	**21**	9	11	13	8	22	19
NEED FOR Punishment	8	4	4	1	3	1	3	2	6
NEED FOR Help	19	***14**	**5**	8	6	2	3	10	9
NEED FOR Agency	2	1	1	0	1	1	0	1	1
YES, program provides	46	**14**	***32**	4	10	18	14	22	24
NO, program does not provide	49	***40**	**9**	20	20	3	6	23	26
UNCLEAR whether program provides	7	3	4	3	0	3	1	6	1
